# Associations Between Perception of Parental Behavior and “Person Picking an Apple From a Tree” Drawings Among Children With and Without Special Educational Needs (SEN)

**DOI:** 10.3389/fpsyg.2018.01613

**Published:** 2018-09-04

**Authors:** Michal Bat Or, Andriani Papadaki, Or Shalev, Elias Kourkoutas

**Affiliations:** ^1^The Graduate School of Creative Arts Therapies, University of Haifa, Haifa, Israel; ^2^Special Education and Psychology, University of Crete, Rethymno, Greece

**Keywords:** parental acceptance-rejection, children, PPAT drawings, special educational needs, gender difference

## Abstract

The present study examines and compares associations between perceptions of parental acceptance/rejection in 191 Greek school age children (84 inclusion class students and 107 typical class students, age range 10–12), and their “Person Picking an Apple from a Tree” (PPAT) drawings. Perception of parental behavior was measured by the “Parental Acceptance-Rejection Questionnaire” (Rohner and Khaleque, 2005). Drawing content was analyzed quantitatively according to a reliable rating system called the Symbolic Content in PPAT drawings (SC-PPAT: Bat Or et al., 2014, 2017). We employed k-means cluster analysis and obtained three relatively discrete PPAT scripts. Drawing content elements and scripts were found to be associated with children’s perceptions of parental behavior; these associations were found mainly among children with special educational needs (SEN) and boys. Results are discussed in terms of children’s subjective experience, clinical implications, and future research directions.

## Introduction

### Children’s Perceptions of Parental Acceptance-Rejection

Research based on a range of different theories has shown consistent and reliable empirical associations between the quality of parental caregiving and child development and psychosocial functioning (Collins et al., 2002), including social competence (e.g., Attili et al., 2010), school performance (e.g., Véronneau and Dishion, 2010), and well-being (e.g., van der Kaap-Deeder et al., 2017). One of the cornerstones of the parent-child relationship is parental warmth and control (for an overview see Maccoby, 2015). In line with this, the IPARTheory (Rohner, 2016) is an evidence-based theory of socialization and development that focuses mostly on the effects of perceived parental acceptance-rejection in childhood (Rohner, 1986, 2015). Parental acceptance is demonstrated through love, affection, care, comfort, support, or nurturance of children, while parental rejection is indicated by the absence or withdrawal of parental warmth, love, or affection (Khaleque, 2015). According to Rohner (1980, 2004), children’s perceptions of parental acceptance-rejection fall within four universal categories: (a) *warmth/affection* – conveyed to the child through physical, verbal, and symbolic parental behaviors; (b) *hostility/aggression* – either physical, verbal, active and/or passive, and problems with the management of hostility and aggression; (c) *indifference/neglect* – a lack of parental concern or interest in the child; and (d) *undifferentiated rejection* – the child’s belief that his/her parent/s do not really care about him or her. While research has relied on parents as the main source of information, there may be gaps between parents’ and children’s perception of parenting behavior (Roe et al., 2006). The child’s subjective perception of parental caregiving can serve as an accurate tool for predicting a child’s behavioral outcomes (e.g., Abar et al., 2015). Numerous studies using diverse research methods have found that children’s perceptions of their family relationships are related to child adjustment (e.g., Grolnick et al., 1991; Cummings and Davies, 1994; Dunn et al., 2002; Ratelle et al., 2004).

Two meta-analyses found that children who perceived themselves as accepted by their parents tended to have socially acceptable behaviors and positive personality characteristics (Khaleque and Rohner, 2012; Khaleque, 2013). At the same time, empirical studies worldwide show a correlation between parental rejection and children’s psychological maladjustment (Miles and Harold, 2003; Putnick et al., 2015); behavioral problems, including conduct disorder, externalizing behaviors, and delinquency (Rohner and Britner, 2002); psychological disorders (Dwairy, 2010); and decreased school performance (Putnick et al., 2015). These findings were consistent regardless of culture, age, and gender (Khaleque and Rohner, 2012). However, children’s subjective experience of their relationship with their parents has implicit and non-verbal aspects (Maier et al., 2004) that are not detected through self-report tools; nevertheless; these perceptions of acceptance-rejection have been investigated mainly through the use of verbal tools only, such as interviews and self-report questionnaires (e.g., Rohner, 2015). Importantly, the child’s experience of parental rejection or indifference is emotionally painful and thus it may be difficult to capture it through direct and explicit ways. Moreover, children with specific cognitive and/or emotional challenges, such as special educational needs (SEN), may also have difficulty verbally expressing their relational experience.

### The Expression of Relational Experience of Children With SEN

SEN students in Greece are identified during the first year of primary school, when most of them (about 70%) require the extra support provided by an inclusion class in order to remain in a regular school and fulfill mainstream educational requirements (Koutrouba et al., 2008). SEN students with severe disabilities are placed in special education schools, and not in inclusion classes. SEN students include children with learning difficulties, learning disabilities (specific or general), ADHD, and/or emotional-behavioral (internalizing and externalizing) problems. Regarding the well-being of parents of SEN students, there is evidence of higher parental stress (e.g., Bonifacci et al., 2014), anxiety, and/or depression levels (Karande et al., 2009). Mothers of children with learning disabilities displayed more avoidance coping behaviors than mothers of typically developing children (Al-Yagon, 2007). Although the expectation might be that SEN students would report higher parental rejection, studies have shown that there was no difference between the self-reports of SEN and non-SEN students (e.g., Bonifacci et al., 2016). This finding can be due to various reasons, some which address the difficulties of SEN students; for instance, children with behavioral problems may experience difficulties in the verbal processing and expression of negative emotions (Hill and Sharp, 2015). Therefore, these children may process and/or express their perceived parental rejection via non-verbal means, for example through the manifestation of behavioral problems that are described in research as outcome variables (Cen and Aytac, 2017). Clinical practice descriptions reveal that children with SEN express painful emotional experiences relating to close relationships through non-verbal activities such as play (Robinson et al., 2017), and expressive art activities (Crimmens, 2006; Bat Or, 2015). We might assume that children with SEN may communicate painful and emotionally laden relational experiences with their parents through their drawings.

### Children’s Drawings as a Mean of Communicating Their Relational Experience

Drawing is a natural activity through which children express and communicate their experiences (Malchiodi, 1998). Thus, children’s drawings have being used to understand children’s subjective experiences in clinical practice (e.g., Linesch, 1994; Ball, 2002) and research (e.g., Gross and Hayne, 1998). McGrath and Carroll (2012) propose that drawing tasks be considered *broadband implicit techniques* (BITs), considering that BIT’s are performance-based tasks that are primarily data-gathering techniques, rather than standardized tests. BITs provide access to mental representations via multiple information channels, including automatic or poorly self-observed mental activities (McGrath and Carroll, 2012). From a psychoanalytic perspective, the content of a drawing, like dreams, conveys multiple meanings (Segal, 1991) and contains manifest as well as latent (hidden) content (Lusebrink, 1990). The images in a drawing can represent thinking, attitudes, emotions, and reflections about human situations and experiences (Milner, 1950; Vass, 2012).

Many studies have demonstrated associations between children’s relational experiences and their drawings; for example, examination of family drawings as representations of attachment in middle childhood confirmed that attachment classifications based on interpretations of combined features of the drawings were related to children’s attachment histories (e.g., Fury et al., 1997; Goldner and Scharf, 2011). Kinetic Family Drawings (KFD: Burns and Kaufman, 1970) were found to represent children’s relational experiences, for example, parental dysfunction as related to alcoholism (Holt and Kaiser, 2001). However, the request to draw a family might be experienced by the individual as too direct, and thus may activate defenses (Kaiser, 1996). One of the solutions for this potential limitation is to ask the individual to draw a subject that would elicit the identified target material indirectly, for example the Bird Nest Drawing (BND: Kaiser, 1996). Accumulating studies of BND drawings show associations between the BND and children’s attachment representations, in particular through aggregations of indicators and global ratings (e.g., Goldner, 2014). However, drawings that contain inanimate objects may also reflect and communicate the child’s relational representations; for example, the house in the House-Tree-Person technique (Buck, 1948; Buck, 1986) was considered to reflect the child’s family relationships and home life, among other issues. Additionally, relationships between drawn objects on a single page are also significant when assessing a child’s sense of subjectivity (Cruz and Feder, 2013).

In the present study, we examined children’s perceptions of parental acceptance-rejection by means of a neutral structural drawing, “Person Picking an Apple from a Tree” (PPAT, Gantt, 1990, Unpublished), which incorporates the theme of a person in the act of reaching a goal (the apple) within a relational context of three objects (person, tree, and apple). Until recently, PPAT drawings were studied mainly for their formal elements, by using the Formal Elements Art Therapy scale (FEATS: Gantt and Tabone, 1998). However, latest PPAT analyses include symbolic content too, using a reliable scoring system entitled the Symbolic-Content rating scale (SC-PPAT: Bat Or et al., 2017). The SC-PPAT was developed according to careful phenomenological observation of PPAT drawing content, which resulted in distilled scales that measure tree characteristics (for example, strength degree), personal features (for instance, degree of activity), and the tree-person relationship (for example, the position of the trunk in relation to the person). Our rationale was that the three objects in the drawing might reflect early relationships; for example, mental representations of the mother-father-baby triangle and the nature of the various cooperative or disruptive alliances within it (Fivaz-Depeursinge and Philipp, 2014). Mental representations of relationships were considered to be scripts, which are sequences of knowledge for given situations/environments that guide the individual’s own expectations and behaviors (Waters and Waters, 2006). In line with this, PPAT drawings of secure individuals (whose security may indicate parental acceptance) tended to depict a cooperative script. In contrast, insecure individuals presented non-coherent script: for example, a person reaching toward a tree whose apples are on the side further away from the person (Bat Or et al., 2015). Exploratory factor analysis of the SC-PPAT scales in a sample of adults (*N* = 215) yielded three main factors: *tree-potency* (the tree’s strength and abundance of fruit, which ranges between high to low tree potency), *person agency* (the degree in which the person is active and successful in reaching the apple), and *tree-accessibility* (the degree in which the tree ‘eases’^1^ the picking process, for instance by bending the tree-truck toward the drawn person) (Bat Or and Ishai, 2016). When the three factors represent a positive direction, the visual script of the drawing reflects reciprocity and promotes a common goal (a successful apple picking). In relation to children’s PPAT, there is no quantitative study, to our knowledge, that associates between relational aspects relating to school age children and PPAT content. A study that examined associations between emotional and cognitive problems of preschool children and their PPAT found gender differences: SC-PPAT scales were found negatively associated in relation to boys’ emotional and behavioral problems as compared to girls’ cognitive problems (Bat Or et al., 2014). The present study aimed to explore school age children’s PPAT and their mental representations of parental relationships. Based on existing literature, we speculated that children who feel rejected by their parents might draw a PPAT script depicting less cooperation between the drawn objects. Gender differences were also analyzed, though we had no specific hypothesis in mind when we began the study.

Our research hypotheses were:

(1) Perceptions of negative parental caregiving will be associated with low reciprocity PPAT scripts, represented by mixed scores on main factors of the PPAT drawings (for example, a low-potent tree with a drawn person demonstrating agency); positive parental caregiving will be associated with a reciprocal and coherent PPAT script (for instance, a potent and accessible tree with a person who has agency).
(2) More associations will be found between perceived parental acceptance-rejection and PPAT drawings among children with SEN than among children without SEN.

## Materials and Methods

### Participants

The sample of 191 Greek fifth and sixth graders (age range 10–12) was drawn from a large research project that included 644 children that were randomly selected from public schools in three prefectures of the island of Crete (Heraklion, Chania, and Rethymnon). Eighty-six percent of the participants were urban residents and 14% semi-urban residents. Since only 13% of the original sample included students from inclusion classes (*N* = 84), we randomly created a matching group (*N* = 107) that reflected a distribution of gender and class similar to that of inclusion class group. The inclusion class group was comprised of 70% boys and 30% girls, so the matching group was similarly constructed and contained 65% boys and 35% girls. N.s differences were found between the two groups in terms of gender and class distribution.

### Instruments

#### Parental Acceptance-Rejection Questionnaire (Child PARQ)

Parental Acceptance-Rejection Questionnaire (Child PARQ) (Rohner, 1990; Adaptation in Greek in Demetriou and Christodoulides, 2006; Giovazolias et al., 2010). The current study used the short form of the Parental Acceptance–Rejection Questionnaire: Child version (*Child PARQ*: Mother version, *Child PARQ*: Father version; Rohner and Khaleque, 2005). The *Child PARQ* short version encompasses 24 items and asks children to interpret their caregiver’s behavior through their own personal experiences. Participants were asked to evaluate each statement on a four-point Likert scale ranging from 1 (*almost never true*) to 4 (*almost always true*). The scales were summed and keyed in the direction of perceived rejection. Mother and Father Child PARQ questionnaires are identical. The Warmth/Affection Scale is composed of eight items, for example, “My father/mother says nice things about me.” Scores were inverted, thus high scores indicate lack of parental Warmth/Affection. The Hostility/Aggression Scale is composed of six statements, for example, “My father/mother hits me, even when I do not deserve it.” The Indifference/Neglect Scale has six items, including statements such as “My father/mother pays no attention to me.” Finally, the Undifferentiated Rejection Scale incorporates four statements such as “My father/mother seems to dislike me.” The Greek Child PARQ was found to be a reliable and valid instrument (Tsaousis et al., 2012; Artemis and Touloumakos, 2016). In the current study, the internal consistency of the total PARQ scores of mothers and fathers in each sub-scale were good (Cronbach’s alphas were 0.853 and 0.851, respectively, *N* = 644).

#### Person Picking an Apple From a Tree” Drawing Task

“Person Picking an Apple from a Tree” drawing task (Gantt, 1990, Unpublished). Although the current study did not used the FEATS scoring system, we followed the instructions proposed by Gantt and Tabone (1998) for administration of the PPAT process. Accordingly, participants were given white sheets of paper (21 by 29.5 cm) and markers in 12 colors (red, orange, blue, turquoise, green, dark green, hot pink, gray, purple, brown, yellow, and black), and were asked to draw “a person picking an apple from a tree” (Gantt and Tabone, 1998). Due to the slightly different composition of colors in the 12 pack markers sold in Greece, the gray-colored marker replaced the magenta color noted in the original Gantt and Tabone (1998) instructions.

*The ’Symbolic Contents in “Person Picking an Apple from a Tree” for school-age children’ (SC-PPAT/c2* Bat Or et al., 2017*)*, comprises nine Likert-scales that range between 0 (the rated feature is absent) and 5 or 6 (the rated feature at its maximum). As can be seen in **Table 1**, the scales measure three central aspects of the PPAT drawing: characteristics of the tree (for example the number of apples on the tree); characteristics of the person (for instance, the degree in which a person is active/passive in the apple picking process); and characteristics of the tree-person relationship (for example, the position of the tree truck in relation to the person).

**Table 1:** Descriptive statistics and interrater reliability for SC-PPAT/c2 scores.

Scale number	Measure	Points on Likert scale	Score number 1	Score number 5 or 6	Mean (*N* = 191)	*SD* (*n* = 191)	Intra-class correlation coefficient (*N* = 64)
1	Quantity of apples on the tree	6	A tree with no apples	A tree with more than 10 apples	4.96	1.43	0.984
2	Strength vs. weakness of tree	5	A very weak tree	A very strong tree	3.65	1.05	0.958
3	The degree to which the person is active/passive in apple-picking	6	The person clearly avoids picking	Extraordinary picking process effort	3.86	1.15	0.903
4	Degree of success in picking the apple	5	No contact between the person and an apple	The person holds one or more apples, disconnected from the tree	2.87	1.40	0.929
5	Contact between person and tree	5	No contact between the person and the tree	Person is contained within the contour of the tree	1.59	0.71	0.986
6	Height ratio between person and tree	6	The person is significantly shorter than the tree (1:5 or more)	The person is taller than the tree (2:1)	3.02	1.27	0.954
7	Position of the tree trunk in relation to the person	5	The tree trunk is clearly inclined away from the person	The tree trunk is clearly inclined toward the person	2.87	0.72	0.958
8	Placement of branches in relation to the person (close vs. far)	5	Branches or treetop are inclined away from the person	Branches are coming out of trunk toward the person	2.80	1.08	0.971
9	The extent to which apples are spread out on the tree either close or far from the person	5	All apples are placed on the side farther from the person	All apples are placed on the side closer to the person	3.34	1.04	0.940

The drawings (*N* = 644) were rated according to the SC-PPAT/c2 rating system; two trained raters coded 10% of each of the drawings, until they achieved substantial agreement. The inter-rater reliabilities were calculated by the Intra-Class Correlation coefficient, which ranged between good and excellent, as can be seen in **Table 1**.

### Procedure

Researchers initially secured approval from the Educational Institute of the Ministry of Education as well as the ethics committee of the University of Crete. Furthermore, meetings were held with the parents of the participants to inform them of the purposes of this research. Parents were asked to sign consent forms. The research was conducted in the schools, and researchers entered the class accompanied by the class teacher. On the first day, the researchers introduced themselves and administered the *Child PARQ*-mother/father questionnaires, and on the second day they administered the PPAT drawing task. Participants were individually asked to draw a person picking an apple from a tree; no time limitation was set. Researchers assured the children that there were no right or wrong answers, and no drawing would be considered an ugly drawing. They informed the children that the questionnaires and the drawings would be collected by the researchers.

## Results

### Descriptive Analyses and Preliminary Analyses

#### SC-PPAT: Descriptive, Factor Analysis, and Cluster Analysis

**FIGURE 1:**
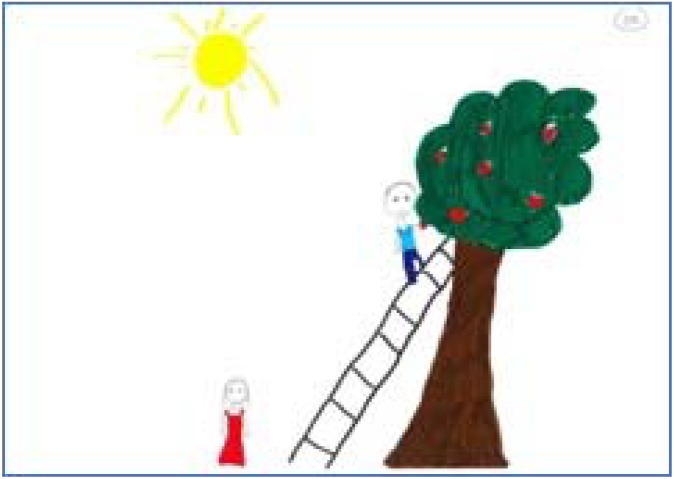
An average PPAT drawing in terms of content elements.

After inter-rater reliability was achieved, the raters coded the remaining drawings individually. As can be seen in **Table 1**, the average drawing in the current study includes a tree with equal strengths and weaknesses that bears five to six apples equally distributed. The tree inclines slightly away from the person, although the branches are neutrally placed in regard to the person’s placement. The person is shorter than the tree (about 1:3), partially active in the picking process, and touches the apple but not the tree. An example for the average drawing can be seen in **Figure 1**. Considering that in this instance Greek children were asked to draw an apple tree, we note that most of the children drew a typical apple tree, in height, proportions, fruit, and form.

Confirmatory Factor Analysis (CFA) was conducted of the original sample *N* = 644 (see **Table 2**) using AMOS software version 23. In comparing the theoretical model (leaning on previous data, Bat Or and Ishai, 2016) and the empirical model, three indices showed good fit; that is, no difference was detected between the two models.

**Table 2:** Confirmatory factor analysis of SC-PPAT/c2 scales.

Measure		Factor	Estimate
Quantity of apples on the tree	<—	Tree’s potency	0.319^∗∗∗^
Strength vs. weakness of tree	<—	Tree’s potency	0.914^∗∗∗^
The degree to which the person is active/passive in apple-picking	<—	Person’s agency	0.719^∗∗∗^
Contact between person and tree	<—	Person’s agency	0.645^∗∗∗^
Position of the tree trunk in relation to the person	<—	Accessibility of tree	0.535^∗∗∗^
Placement of branches in relation to the person	<—	Accessibility of tree	0.591^∗∗∗^

*χ^2^ (7) = 11.94, *p* = 0.103, CFI = 0.98, NFI = 0.96, RMSEA = 0.033 [90% CIL.00-0.06]. ^∗∗∗^*p* < 0.001*.

As shown in **Table 2**, three main factors were obtained, each consisting of two scales. ‘Person’s Agency’ pertains to the drawn person’s activity/passivity in the apple picking process, and contact level between person and tree. ‘Tree Accessibility’ pertains to the tree’s orientation toward the drawn person, including inclination of the tree trunk, and placement of branches in relation to the person. Finally, ‘Tree Potency’ pertains to the characteristics of the tree, including its strength, and the number of apples it bears. These factors yield a total of 68% of the explained variance.

Inter-factor associations were also measured for our sample (*N* = 191), showing only one medium positive association between the drawn ‘Person’s Agency’ and the ‘Tree’s Accessibility’ (*r* = 0.249, *p* < 0.001). Specifically, the stronger the person’s agency in the apple picking process, the more accessible the tree is to the person.

**FIGURE 2:**
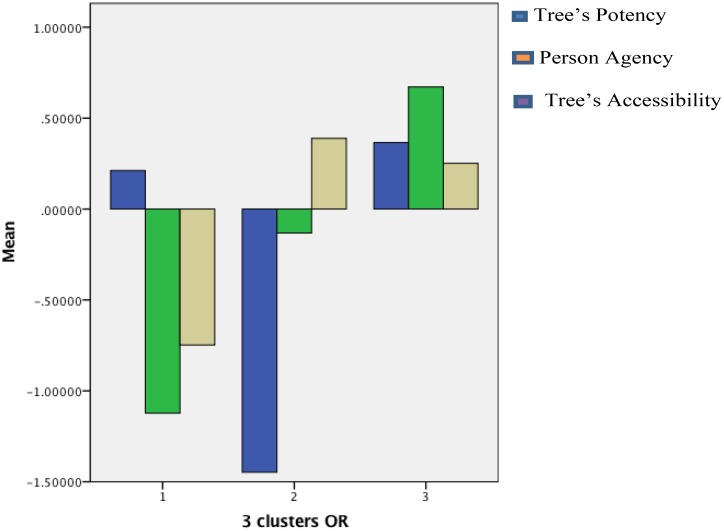
Bar graph representing the three clusters in terms of Z scores.

After obtaining the three factors, we subjected the data to k-means Cluster Analysis (*N* = 191) to generate relatively discrete clusters of PPAT narrative. Drawings were grouped according to the magnitude of the main factors scores within different combinations of main factors. **Table 3** describes the clusters’ centers in terms of the main factors of the drawings, and **Table 4**, the one-way ANOVA showing significant differences between the three main factors in each cluster. The significance of **Tables 3** and **4** can be best illustrated by the bar graph in **Figure 2**, which represents the three clusters in terms of the Z scores of three PPAT drawings scripts. These scripts will be detailed, together with accompanying drawings, below.

**Table 3:** Final cluster centers.

	Cluster		
PPAT’s main factors	A	B	C
Potency of tree	4.63	2.89	4.86
Agency of person	1.83	2.85	3.22
Accessibility of tree	2.39	3.07	3.00
N	55	47	89

**Table 4:** One-way ANOVA for factor differences within the three clusters.

		Sum of squares	df	Mean square	*F*	Sig.	η^2^_p_
Tree’s potency	Between groups	130.032	2	65.016	209.556	<0.001	0.05
	Within groups	58.328	188	0.310			
	Total	188.361	190				
Person’s agency	Between groups	68.645	2	34.322	113.205	<0.001	0.66
	Within groups	56.999	188	0.303			
	Total	125.644	190				
Accessibility of tree	Between groups	9.559	2	4.780	9.485	<0.001	0.18
	Within groups	94.735	188	0.504			
	Total	104.295	190				

**FIGURE 3:**
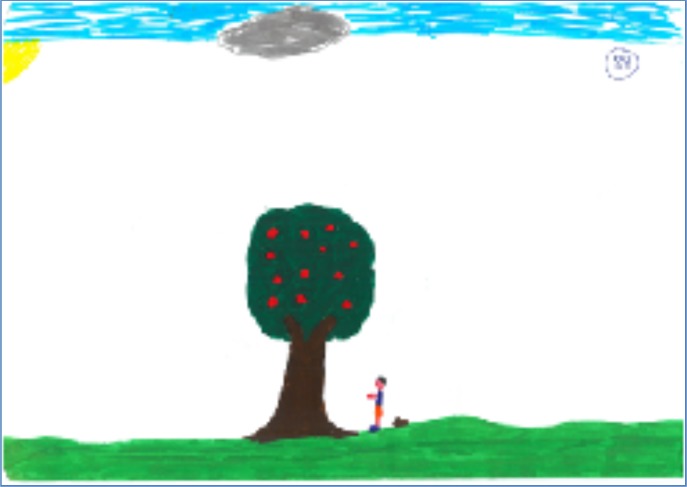
Cluster A drawing: “*Not joining”* script.

**FIGURE 4:**
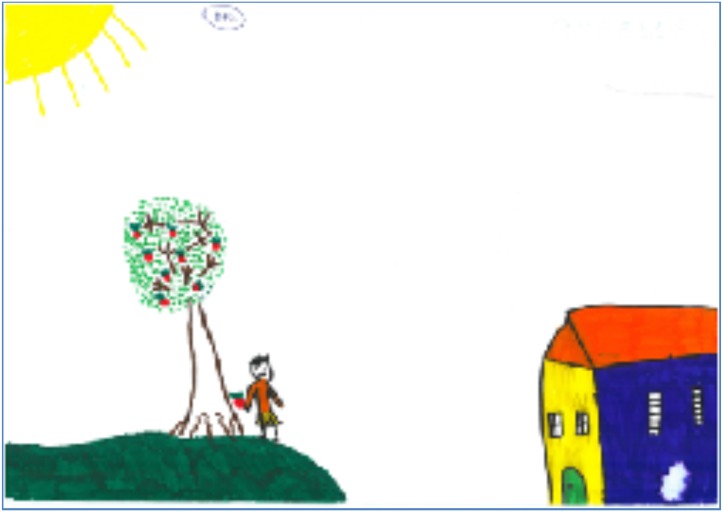
Cluster B drawings “*Moderate efforts”* script.

**FIGURE 5:**
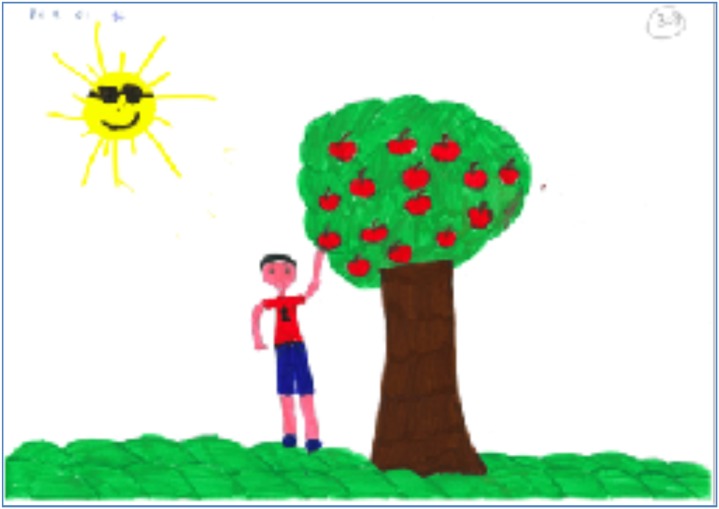
Cluster C drawings “*Reciprocity and actualization”* script.

To summarize, three clusters were identified: **Figure 3** illustrates the cluster A drawing (*n* = 55) comprised of a potent tree (strong and abundant in fruit) but not accessible (for example, inclining in the opposite direction) with a person with low agency (for example, a passive figure). This cluster was labeled “*Not joining”* because the tree and the person are not synchronized in their positions/motions in relation to the apple picking. **Figure 4** illustrates the script in cluster B drawings (*n* = 47), composed of a non-potent tree (for example, weak with only a few apples), neutral in accessibility (a bit more accessible than in cluster A, however, tree trunk is upright), and a person with medium agency (partially active in the picking process); this cluster was labeled “*Moderate efforts.”* Finally, cluster C drawings (*n* = 89) depicted a reciprocal script with a potent and accessible tree, and a person with high agency (see **Figure 5**). This cluster was labeled “*Reciprocity and actualization.”* In terms of the PPAT script, cluster C describes the most coherent scrip in relation to the reciprocity of the drawn objects, while clusters A and B reveal non-coherent scripts and lower reciprocity between the drawn objects.

**Table 5** presents the range of scores, means, and standard deviations, as well as minimum and maximum scores for each PARQ category. The descriptive statistics reveal that on average, children reported lower perceived parental rejection, as manifested in low scores on Hostility/Aggression, Indifference/Neglect, and Undifferentiated/Rejected scales. The Warmth/Affection and Lack of Parental Warmth/Affection scales were inverted. Low to medium correlations (r range 0.266–0.710, *p* < 0.001), were found in the scores of fathers and mothers, thus showing similarity and differences between the perceptions of mothers and that of fathers.

**Table 5:** Descriptive statistics of children’s perceptions of paternal and maternal PARQ subscale scores.

	Minimum	Maximum	Mean	*SD*
Father lack of warmth/affection	8	32	11.92	3.99
Father hostility/aggression	6	24	8.05	2.72
Father indifference/neglect	6	24	9.80	3.15
Father undifferentiated/rejected	4	15	5.57	2.01
Mother lack of warmth/affection	8	32	10.62	3.72
Mother hostility/aggression	6	24	7.87	3.23
Mother indifference/neglect	6	24	8.98	3.08
Mother undifferentiated/rejected	4	16	5.62	2.37

Preliminary analysis using the Independent-Sample *T*-test revealed that child’s age had no effect on perceived parental acceptance-rejection components. We employed MAVOVA to determine if child’s gender and the type of class (inclusion class vs. typical class) were significantly related to perceived parental acceptance-rejection components (for mothers and fathers separately). MANOVA revealed no differences.

*Gender, age, and class type differences and associations with PPAT drawings:* Independent-Sample *T*-tests revealed that the child’s age had no effect on the drawing. We used MANOVA to determine if child’s gender and class type were significantly related to the PPAT drawings’ main factors and found no significant difference.

### Hypotheses Testing

#### Associations Between Perceived Parental Acceptance-Rejection and Content of PPAT Drawings

We first describe the results for the whole sample, and then results for gender groups. The associations were calculated in two ways: Pearson correlations between main factors of PPAT drawings and the criterion variables, and then associations related to parental acceptance-rejection in terms of the three clusters of drawings.

Pearson correlations between the main factors of PPAT drawings and criterion variables showed one significant negative association between the drawn person’s agency and perceived maternal hostility/aggression for the whole sample (*r* = -0.278, *p* < 0.001). This means that the more the child perceived her/his mother as hostile/aggressive, the less the drawn person was active and touched the tree, or, in other words, less competent in picking the apple. We also analyzed the first hypothesis as related to PPAT’s three clusters. One-Way ANOVA showed a significant difference between clusters A and C in terms of the children’s perceptions of maternal Hostility/Aggression: *F*(2, 169) = 4.00, *p* = 0.020. *Post hoc* analyses found that children who reported their mother as more hostile/aggressive drew a PPAT that suggested a “*Not joining”* script (a potent but less accessible tree, and a person with low agency) in comparison to children who perceived low maternal Hostility/Aggression. Specifically, the latter drew a script of “*Reciprocity and actualization”* (a potent and accessible tree, with a person with agency).

We further examined these associations in relation to gender. After dividing the sample into groups of boys and girls, we found, – after applying the Bonferroni correction – two significant negative associations between perceived parental acceptance-rejection and PPAT drawings among boys only (*n* = 129). Specifically, the more boys perceived their mothers as hostile/aggressive, and/or as lacking in warmth, the more the drawn person in PPAT lacked agency (*r* = -0.340, *p* < 0.001; *r* = -0.265, *p* = 0.004 accordingly).

In terms of cluster analysis, we found a significant difference between Clusters A and C relating to maternal hostility among boys only. Specifically, a one-Way ANOVA showed significant differences between clusters A and C in terms of the children’s perceptions of maternal Hostility/Aggression: *F*(2, 111) = 6.32, *p* = 0.002. *Post hoc* analyses revealed that boys who reported their mother as being more hostile/aggressive drew a PPAT that indicated a “*Not joining”* script; in comparison, boys who reported low maternal Hostility/Aggression drew a script of “*Reciprocity and actualization*.” These results confirm our first hypothesis; however, they also indicate gender differences, namely, that for the most part, associations are found between parental acceptance-rejection and PPAT mainly among boys.

### Comparison of the Associations Between Perceived Parental Acceptance-Rejection and PPAT Drawings’ Content Among Children With and Without SEN

After splitting the sample according to classroom types, six significant associations were found between criterion variables and the PPAT drawings of children with SEN, while n.s associations were found among children without SEN. After a Bonferroni correction, two negative associations were found between ‘Person’s Agency’ and maternal Hostility/Aggression (*r* = -0.418, *p* < 0.001), and paternal Hostility/Aggression (*r* = -0.324, *p* < 0.001); In addition, a One-Way ANOVA showed significant differences between clusters B and C in association to perceived paternal Indifference/Neglect: *F*(2, 74) = 3.52, *p* = 0.035. In specific, children with SEN who scored high on paternal Indifference/Neglect tended to draw a PPAT that indicated a script of “*Moderate efforts”* as compared to children with SEN that scored low on paternal Indifference/Neglect and tended to draw a PPAT that suggested “*Reciprocity and actualization.”* These results confirm our second hypothesis.

## Discussion

The aims of the present study were twofold: to explore associations between children’s perceptions of their parents’ behavior toward them and their PPAT drawings, and to examine these associations in relation to classroom type. Analyses of the findings revealed also gender differences. We hypothesized that associations would be found between perceived parental behavior and PPAT content/script; specifically, positive parenting would be related to positive contents and a reciprocal script, and perceptions of negative parental caregiving would be related to PPAT drawings with negative contents and low reciprocity scripts. Associations between children’s perceptions of their parents’ behavior and PPAT drawing content were found mainly among boys and among children with SEN (or both).

In the whole sample, we found one association between children’s perceptions of maternal Hostility/Aggression and the drawing of a person with lower self-agency; nevertheless, further analysis revealed that this association was present among boys only. For this reason, we first discuss gender differences and then discuss the comparison between children with and without SEN. After discussing main findings, limitations and research suggestions, clinical implications are presented.

### Associations Between Perceptions of Parental Acceptance-Rejection and the PPAT Drawings of Boys

The present study found that the more boys perceived their mothers as hostile/aggressive, or lacking in warmth, the more the drawn person in their PPAT tended to show lower agency (less active and having limited contact with the tree). No associations were found in the girls’ group. This result was further strengthened by using the cluster analysis method to discern between visual scripts: boys who perceived their mothers as most hostile/aggressive tended to draw a non-reciprocal script, which we called a “*Not joining”* script; in contrast, boys who reported the lowest scores in maternal Hostility/Aggression tended to draw “*Reciprocity and Actualization”* scripts. Based on developmental norms of 10–12 years old children, their drawings are expected to display intellectual realism (objects drawn are recognizable); however, children of this age still lack the ability to draw visually realistic figures/images (Cox, 1993). Most children from the age of 8 are able to depict a human figure in action (Goodnow, 1978). These norms may further validate our findings, which revealed a link between the child’s relational perceptions and the drawing of human figures that display less agency in the picking process.

Parental caregiving and the nature of the child-parent relationship shape the child’s internal working models (Grossmann et al., 2006) which in turn determine personal expectations from the outside world, level of trust and sense of safety (Semrud-Clikeman, 2007). Parental acceptance is associated with higher self-esteem of children in their middle childhood (Bornstein, 2002). Furthermore, there is empirical evidence that parents who exhibit high levels of aggression and hostility toward their child are perceived by their child as threats and sources of insecurity (Grych and Fincham, 1990; Davies and Sturge-Apple, 2007; Repetti et al., 2011). Parental behaviors of this kind can hinder the child’s ability to form cooperative relationships (Semrud-Clikeman, 2007; Rudolph, 2009). This can also be explained by a model of emotional intelligence that involves the ability to perceive, understand, and regulate emotions (Mayer and Salovey, 1997). One mechanism through which children learn to manage their own emotions is by modeling the way their parents express and regulate emotions (Morris et al., 2017). On the subject of parental hostility, traditional Greek culture values family loyalty and adherence to group norms (Zervides and Knowles, 2007), and this is linked to controlling child rearing practices (e.g., Papps et al., 1995). We may thus speculate that parental hostility would be associated with lower emotional intelligence and compromised social abilities among children, and these might be reflected in a PPAT drawing that displays a non-joining script.

The results of this study pertaining to the boys’ group correspond with findings from Bat Or and Ishai (2016) that showed that PPAT drawings of insecure adults represent less positive and less reciprocal relationships between the drawn objects in comparison to PPAT drawings of secure adults. Moreover, PPAT drawings of boys that reported lower levels of maternal hostility/aggression depicted a coherent script including both a strong and accessible tree with more apples on it and a drawn person who was more active in the picking process. This may reflect the child’s inner script of joining and reciprocity.

Yet, the question still remains as to why we found associations only among boys, and not among girls. We suggest that there may be a salient gender difference in children’s emotion expression. As a result of gender socialization, the verbal narratives of girls and women are more emotionally laden than those of boys (Fivush, 2007). Specifically, parents use a larger vocabulary of words pertaining to emotions when speaking to their daughters than when conversing with their sons (Fivush, 2007). According to Brody’s (1999) theory of gender differences, parents and other socialization agents may respond to boys in ways that dampen and limit emotional expressiveness. Thus, gender socialization may provide girls with more opportunities for emotional discourse than boys (Melzi and Fernández, 2001). It could be speculated that since boys are more restricted in verbal expressions and in sharing their negative emotional experiences than girls, they could communicate their subjective experiences, especially negative emotions, through different means of non-verbal communication, such as physical aggression (see the meta-analysis of Card et al., 2008). In line with this, empirical evidence indicates that boys are more sensitive than girls to harsh physical punishment by parents, as demonstrated by conduct-related problems (Berzenski and Yates, 2013). Additionally, the observed gender difference may be attributed to common Greek sexual stereotypes, especially those related to emotional expression: fearful that they will be considered less masculine, boys tend to show less vulnerability than girls (e.g., Makri-Botsari and Karagianni, 2014).

In light of these findings, we can conjecture that the PPAT drawings allowed the boys to express indirectly their perceptions regarding emotional ties with their parents, and/or their sense of agency, without having to engage in verbal communication (White et al., 2004). This may also emphasize the particular link between perceived maternal hostility and lack of warmth of boy’s mental representations.

### Association Between Perceptions of Parental Acceptance-Rejection and PPAT Drawings Among Children With and Without SEN

The results of this study indicate associations between perceptions of parental acceptance-rejection and PPAT drawings among children with SEN; specifically, between perceived paternal and/or maternal hostility/aggression and a drawn person with lower self-agency. In addition, the child’s perception of paternal neglect was associated with the “*Moderate efforts”* script, while the lowest scores on paternal neglect were associated with the “*Reciprocity and actualization”* script. No association was found among children without SEN.

The results provide two main insights: the first is that the PPAT drawings of children with SEN revealed stronger affinity to their perceptions of parental acceptance-rejection than the PPAT drawings of children without SEN. Since children with SEN have cognitive and/or emotional-behavioral problems, and/or impaired verbal and non-verbal information processing abilities, we may speculate that the PPAT drawing serves as a channel to process/express parental hostility, neglect, or rejection. Children with SEN have an exceptional and cardinal need for family connectedness and support because they struggle with negative developmental outcomes (Cen and Aytac, 2017), and suffer from affiliate stigma (Banga and Ghosh, 2017). In such cases, they would be strongly affected by parental hostility, neglect, and rejection. Associations found among these children may indicate that children with SEN who experience parental rejection internalize the experience as a mental script in which the underachieving self has lower expectations of cooperation and future success. Regarding associations between paternal neglect and the PPAT scripts, it may be that children expressed their experience of paternal neglect via the drawing’s script, where there is either no help (the non-accessible tree), or insufficient actions taken to reach the goal (the partially active human figure), or a lack of resources (the weak tree, with less apples on it). This visual script may serve to highlight a system of relationships that lack mutuality and collaboration.

The second contribution of this study is that it revealed associations between perceptions of father and PPAT drawings solely among children with SEN. Empirical and clinical research show that parenting children with SEN is much more complex because the child’s special needs are a source of parental stress (Bonifacci et al., 2016) and a subjective burden (Banga and Ghosh, 2017). Since mothers are the primary caregivers in most families, they assist children with SEN with their homework as a daily activity, which may be potentially stressful for mothers and children alike (Bonifacci et al., 2016). The father may serve, in cases of maternal stress, as a protective agent, and add his unique view and support to the triangle (Fivaz-Depeursinge and Philipp, 2014). In line with this, in a recent study, children with specific learning disabilities showed higher preference of fathers (Bonifacci et al., 2016). However, when the father is overwhelmed, hostile, irritated, or neglectful, the child may feel deeply abandoned, unloved, and unworthy. Associations found in the present study among children with SEN may reflect the crucial role that a father plays in the child’s mind. Further research is needed to examine paternal impact on children with SEN.

### Limitations and Directions for Future Research

The present study has some methodological limitations. First, the combination of drawings and self-reported questionnaires is problematic in terms of theoretical validity, since each method (verbal vs. non-verbal) may communicate different representational levels (Bosson et al., 2000; Andreou and Bonoti, 2010). Further to that, while theoretical concepts measured by a self-report questionnaire focus on specific mental phenomena, projective drawings contain multichannel information (McGrath and Carroll, 2012). In a future study, it may be worth measuring children’s experience through interviews that capture respondent’s defenses, affects, and less conscious layers. In addition, future research should encourage children to provide a verbal narrative for their PPAT so that children’s interpretations of their own drawings can also be considered. This may contribute to further understanding their relational perceptions (Matsopoulos et al., 2017). Secondly, although social networks expand significantly in middle childhood (Bornstein, 2002; Blake, 2008), and children spend less time with family members and more with peers and other adults outside of the family, our study did not include children’s perceptions of other people close to them. We thus encourage future studies to investigate these perceptions and their association with PPAT drawings; one possible subject is the child’s relationship with her/his teacher. Thirdly, in light of previous findings that indicate significant associations between cognitive disfunctions and PPAT drawings among preschool children (Bat Or et al., 2014), we recommend that a future study include measures of cognitive abilities, in order to control their possible impact on PPAT pictorial content among middle childhood children. In addition, the levels of SEN were not addressed, although they could have an impact on the PPAT drawings in terms of problem solving. And lastly, we must bear in mind assertion Gantt’s (2004) that the PPAT drawing captures an emotional/clinical state rather than assesses personality. Accordingly, an additional limitation of this study may relate to the possible impact of parent-child interactions that occurred the morning of the PPAT administration.

### Clinical Implications

The present study examined primary school age children, who, being in the latency stage, tend to be less verbal in communicating their experiences and perceptions regarding their attachment relationships to significant others (Blake, 2008). This underscores the importance of using a non-verbal method in the form of art-based tasks so that the clinician can learn and understand the child’s subjective experience, even more so, for children with SEN (Kourkoutas et al., 2014). As they are familiar playful tasks, drawings may serve the child and clinician in the exploration of the child’s inner landscapes. The current study reveals associations between PPAT drawings of school age children and their experiences with their parents. Clinicians are thus encouraged to carefully observe the child’s drawing and pay attention to the drawings’ script (reciprocity and actualization, moderate efforts, and not-joining), rather than searching for single indicators. However, before establishing associations to parental relationships by means of a straightforward “dictionary approach,” (Gantt, 2004), the clinician must also consider other aspects, for instance cognitive dysfunctions, motivation, and the alliance between therapist and the child. When examining perception of parental aggression or rejection, we need to take the child’s sensitivity into account. Feelings of rejection from a parent may be painful, and hard to express due to shame and self-blame (Harter, 2015). The PPAT drawing task may provide a secure space for exploration of non-reciprocal or excluding relationships. The current study emphasized the possible imprint of parental aggression on the child’s mind. Intervention in these cases are crucial, considering that aggressive behavior in the child’s family is one of risk factors for psychological problems in childhood and adulthood (Repetti et al., 2011). **Therapeutic changes might be reflected by changes in PPAT drawings scripts. Research is recommended for further exploration of these possibilities.**

### Conclusion

The present study has exemplified that factor analysis and clustering methods provide a reliable means of examining the main contents of the drawings, and discerning specific scripts that may be related to the child’s relational experience. Parental rejection components were found associated to lower agency of the drawn person, and to non-coherent and non-reciprocal PPAT drawing scripts; in comparison, children that reported on the lowest parental rejection components (meaning parental acceptance components) drew coherent and reciprocal PPAT drawing scripts.

These drawn scripts might be representative of the children’s internal working models, and thus influenced by their relational expectations, i.e., their hope to receive assistance from other people, how cooperatively they interact, their self-worth, and their ability to achieve goals (Grossmann et al., 2006). The present study confirms that a broader observation of drawing narratives/script is required to understand the child’s subjective relational experience. This is similar to clinical work with clients, where clinicians attempt to gain access to the client’s relational scripts through personal narratives (McLeod, 1997). In addition, differences found in relation to gender and SEN underscore the importance of contextual factors in understanding children’s drawings.

## Ethics Statement

This study was carried out in accordance with the recommendations of the Educational Institute of the Ministry of Education ethics committee. The protocol was approved by the Educational Institute of the Ministry of Education ethics committee. All parents of subjects gave written informed consent in accordance with the Declaration of Helsinki.

## Author Contributions

MBO together with her group of colleagues have developed the SC-PPAT rating system, and led the process of rating and analyzing drawings. AP Initiated and coordinated the research project for her Ph.D. research. OS assisted in further refining the rating tool, and focused on the drawings and PARQ for her MA thesis dissertation. EK supervised the whole research.

## Conflict of Interest Statement

The authors declare that the research was conducted in the absence of any commercial or financial relationships that could be construed as a potential conflict of interest.

## Footnotes

1 It is mostly slightly easing, such as a gentle cooperative gesture, and not a significant rotation, that was described by Gantt and Tabone (1998), which characterizes pathology.
